# Habitat mediates coevolved but not novel species interactions

**DOI:** 10.1098/rspb.2021.2338

**Published:** 2022-01-12

**Authors:** Joshua P. Twining, Chris Sutherland, Neil Reid, David G. Tosh

**Affiliations:** ^1^ Department of Natural Resources, Cornell University, Fernow Hall, Ithaca, NY 14882, USA; ^2^ School of Biological Sciences, Queen's University Belfast, 19 Chlorine Gardens, Belfast BT9 5DL, UK; ^3^ Institute of Global Food Security (IGFS), Queen's University Belfast, 19 Chlorine Gardens, Belfast BT9 5DL, UK; ^4^ Centre for Research into Ecological and Environmental Modelling (CREEM), The Observatory, Buchanan Gardens, University of St Andrews, St Andrews, Fife, KY16 9LZ, UK; ^5^ National Museums NI, 153 Bangor Road, Cultra BT18 0EU, UK

**Keywords:** habitat complexity, interspecific interactions, occupancy, native predator, invasive species, multi-species models

## Abstract

Ongoing recovery of native predators has the potential to alter species interactions, with community and ecosystem wide implications. We estimated the co-occurrence of three species of conservation and management interest from a multi-species citizen science camera trap survey. We demonstrate fundamental differences in novel and coevolved predator–prey interactions that are mediated by habitat. Specifically, we demonstrate that anthropogenic habitat modification had no influence on the expansion of the recovering native pine marten in Ireland, nor does it affect the predator's suppressive influence on an invasive prey species, the grey squirrel. By contrast, the direction of the interaction between the pine marten and a native prey species, the red squirrel, is dependent on habitat. Pine martens had a positive influence on red squirrel occurrence at a landscape scale, especially in native broadleaf woodlands. However, in areas dominated by non-native conifer plantations, the pine marten reduced red squirrel occurrence. These findings suggest that following the recovery of a native predator, the benefits of competitive release are spatially structured and habitat-specific. The potential for past and future landscape modification to alter established interactions between predators and prey has global implications in the context of the ongoing recovery of predator populations in human-modified landscapes.

## 1. Introduction

Determining the mechanisms underpinning species occurrence and how perturbations can alter species coexistence and biodiversity patterns is a fundamental goal in ecology. Although typically viewed as pairwise, species interactions are embedded within complex multi-trophic networks. Outcomes of interactions cannot be understood without considering the indirect interactions resultant from the presence of extra-pair predators, pathogens, or prey [1], and simplifying systems to pairwise interactions necessarily omits important complexities posed by real world systems [2]. The planetary-scale influence of human activity has brought into sharp focus the need to predict how whole communities respond to multiple anthropogenically driven stressors. This requires an explicit focus not only on how specific species respond to change, but also how interactions and interdependencies among species are affected by changing environments.

Invasive species have been associated with increased vertebrate extinctions more than any other factor [3,4] and provide compelling examples of how novel indirect interactions can alter established species interactions, with potential outcomes ranging from complete exclusion and species extirpation to fugitive coexistence [5]. For example, in Great Britain and Ireland, landscape-scale declines and extirpation of native red squirrels (*Sciurus vulgaris*) results from disease-mediated competition with invasive North American grey squirrels (*Sciurus carolinensis,* [6]), the reservoir host of the squirrelpox virus, a fatal pathogen to the native red squirrel.

Despite populations being globally depleted, far below natural levels owing to human persecution, certain native predator populations, both large and small bodied, are recovering in response to protective legislation and conservation efforts [7,8]. Native predators are returning to landscapes that have been greatly altered since their extirpation, both through human modification of habitats and through the introduction of non-native species. Emerging research suggests that native predator recovery has the potential to benefit native prey populations indirectly through biological control of naive invasive prey/competitor species over spatial scales meaningful to the conservation and management of wildlife populations [9–11]. However, the generality of such predictions remains equivocal owing to the presence of indirect interactions between species, and the heterogeneity exhibited by most landscapes that novel interactions occur in.

Heterogeneity in the structure and configuration of habitat can mediate predator–prey interactions through its influence on the density and type of functional response exhibited by predators [12,13]. Habitat is a determining factor in the hunting capacity of predators, and the ability of prey to detect, avoid, or escape predators [14,15]. Changes in habitat complexity through human modification could hypothetically alter the outcome of species interactions through altering the functional response, attack rate, and handling time of predators [16]. Thus, extrapolating inference about species interactions from one position on an environmental gradient to another, where the densities or functional responses of species are different, could lead to unexpected outcomes.

Despite the relevance of habitat in mediating indirect species interactions in the face of ever-increasing global change, empirically demonstrating the influence of habitat on predator–prey interactions is quantitatively challenging, particularly when focusing on wide ranging, low density and elusive vertebrate predators. Manipulative experiments at characteristic spatial scales are typically implausible within the strictures of research funding and longevity, and thus, evidence is often observational, based on natural landscape-scale investigations such as those presented by asynchronous predator recovery dynamics (e.g. [10,17]). The standard of evidence required to inform policy is necessarily high and, thus, appropriate data collection and associated modelling techniques that, for example, explicitly account for species interactions and imperfect detection are essential.

The difficulties of making robust predictions about novel species interactions in an applied context are compounded in landscapes which are modified by human activity and display pronounced spatial heterogeneity, as is typical of contemporary landscapes across the globe. Without sufficient understanding of the role of habitat in mediating predator–prey interactions, conservation policies focused on ecological recovery and restoration, including the reintroduction of predator populations, could result in unintended, adverse consequences for native prey. The consequences of failing to predict novel species interactions are exemplified by numerous ill-fated attempts to introduce non-native generalist predators as biological control agents to island ecosystems, leading to disastrous impacts on naive native prey species, often resulting in severe decline, extirpation, or extinction [18].

Here, we investigate the role of habitat in mediating the impacts of the recovery of a native predator, the pine marten, on native red and invasive grey squirrels in Ireland; two species that are linked through competition and pathogen-mediated apparent competition. We use multi-species occupancy models applied to a dataset collected on three occasions over 5 years from 2015 to 2020 to examine whether species co-occurrences and interactions differ along environmental and spatial gradients. We expect: (i) the impact of the pine marten on grey squirrels to be consistent regardless of local habitat owing to the naivety of the invasive species to the native predator [19]; (ii) the interactions between the pine marten and the red squirrel to be dynamic and dependent on habitat, with more structurally complex and diverse habitats resulting in lower impacts on the native prey species; and (iii) the competitively linked native-invasive prey species interactions to be mediated by habitat [20].

## 2. Methods

### (a) Multi-species surveys

A survey spanning a 5 year period documenting the occurrence of pine marten and grey and red squirrel was conducted throughout Northern Ireland between 2015 and 2020. The survey was repeated three times, initially in 2015 with 332 sites surveyed by citizen scientists provided with camera traps and trained for their consistent use (for full Methodology, see [11]). This survey was repeated in 2018 with 172 sites, and in 2020 with 207 sites using the same methods. At each site, a single camera trap was deployed at a point randomly selected by the surveyor within an independent 1 km grid. Cameras were installed at head height on a tree overlooking a wooden squirrel feeder erected on an adjacent tree. Feeders were baited with peanuts and sunflower seeds in 2015 and 2020, but just sunflower seeds in 2018. Cameras were set to take three images per trigger with a 1 s reset time. Camera traps were deployed for 7–14 days at each location (mean = 10.3 days) after which cameras were retrieved for data extraction and species identification. Detection records were created for each species over the recording period. Only one detection was allowed per species for each 24 h period of sampling to ensure independence. Any variation in survey effort (duration of camera deployment) was recorded and accounted for during analysis. A map of the sampling sites, and the makes and models of the cameras and the settings used in this study are reported in the electronic supplementary material, appendix S3.

### (b) Occupancy modelling

Our focus is on estimating the co-occurrence of pine marten, red squirrel and grey squirrel, which we do using a hierarchical modelling framework, specifically, the recently developed multi-species occupancy model for interacting species [21]. This approach extends the standard occupancy model [22], that accounts for imperfect detection using a repeat visit sampling design, to include an explicit component for how species interact, including modelling these interactions as a function of covariates.

To explain variation in marginal occupancy rates (the occupancy of a species in the absence of the effects of other species) and conditional occupancy rates (the occupancy of a species conditional on the presence of another species), we considered six landscape variables that had previously been observed to influence the three species [11,23]. These variables were related to forest composition (%broadleaf woodland; %coniferous plantation), human disturbance (number of people per km^2^; %urban and suburban land cover) and non-forested and aquatic habitat conditions (%heath; river and stream density). For details on the mean and variation of covariates, see the electronic supplementary material, table S3.1. We controlled for potential geographical variation in occupancy by including latitude and longitude (and their interaction) of camera trap sites as covariates. We divided Northern Ireland into 14 402 1 km^2^ grid squares and each covariate was summarized at the 1 km^2^ scale. A 1 km^2^ resolution was selected for two reasons: (i) it approximates the home range size of a female pine marten, being the largest of the three species [13]; and (ii) it is a typical intuitive map scale frequently used at regional and national scales. It is true that squirrels have smaller home ranges (e.g. [24]), but this was deemed less important as one of the fundamental assumptions of the models used is that of independence. Therefore, by ensuring independence of the species with the largest range, then independence is met for the other two species with smaller home ranges. The values for each camera site were the values for the grid within which they occurred. To explain variation in detectability, we considered three observation covariates. These were bait type (sunflower seeds and peanuts in 2015 and 2020 versus sunflower seeds only in 2018), a behavioural response (1 if the focal species had been observed previously, 0 if not), and the sampling occasion (ranging from 7 to 14, where a sampling occasion is 1 day). All continuous covariates were scaled and standardized to have unit variance and a mean of zero, and, based on variance inflation factors, there was no evidence of collinearity between any covariates (e.g. [25]).

The core of the co-occurrence model is a state model for estimating latent state of a site (*ψ*), where, if *s* is the number of species, the possible states are the (2*^s^* − 1) possible combinations of species. For example, if there are two species, the possible states are *Z* = ([00], [01], [10], [11]), and *ψ_i_* is the probability of being in the *i* = 1, 2, 3, 4th state. Here, the *ψ*'s are assumed to be multivariate Bernoulli random variables and can also be modelled as a function of covariates. Importantly, each state is first order if occupied by single species, second order if occupied by two species and so on up to order *S*, and each combination can be modelled using standard linear modelling. This means that covariate models can be constructed for species-specific occupancy (first order) and for pairwise interactions (second order) to investigate how species occupancy responds to interspecific (other species) and environmental factors. For example, using this approach, Rota *et al*. [21] found that coyote (*Canis latrans*) occupancy increased with disturbance in the absence of bobcats (*Lynx rufus*), but decreased when bobcat was present, highlighting how species interactions can vary in response to environmental gradients.

Here, we specify first-order models for each species based on results from single-species occupancy models. For each species, we considered all additive combinations of the eight occupancy covariates to describe variation in occupancy (see above), and all additive combinations of the three observation covariates and six landscape covariates to explain variation in detection probability (see above). We note also that these data were collected across three primary survey periods (2015, 2018, 2020), and because the focus was not in estimating colonization–extinction dynamics (which would be unadvisable with only 3 years of data), we used a ‘stacked’ design whereby each site–year combination was treated as a distinct site. As such, we include a year effect in all models to account for any non-independence. Temporal replication between years was limited (see the electronic supplementary material, figure S3.1), thus it was not possible or necessary to fit a site effect on the models. Using the secondary stage approach [26], we first used Akaike information criterion (AIC) to find the most parsimonious covariate combination for detection probability keeping occupancy constant (i.e. *ψ*(.)), and then, keeping detection constant (i.e. *p*(.)), used AIC to determine the most parsimonious covariate combination for occupancy. Parameter redundancy was evaluated following Arnold [27] such that the parameters that were included but resulted in less than −2 AIC units from the next best model were considered uninformative and removed. The single-species analysis was conducted in R v. 3.6 [28] using the package *unmarked* for model fitting (function *occu()*) and goodness-of-fit testing (*parboot()*), the latter showing no issues with model fit [29], and AIC-based model ranking was conducted using the package *MuMin*.

The combination of the AIC-best models for each model component was used to specify the species-specific (i.e. first order) models in the multi-species model (table 1). Specifically, the top pine marten (PM) model included %coniferous plantation, %broadleaf forest, %urban, and latitude, with detection varying by the bait used, the occasion number, a behavioural response, and %coniferous plantation. The top red squirrel (RS) model included %coniferous plantation, %urban, and latitude, with detection varying by the human population density, a behavioural response and %broadleaf forest. The top grey squirrel (GS) model included %coniferous plantation, %broadleaf forest, %urban, and both latitude and longitude, with detection varying by the bait used, a behavioural response, the %broadleaf, %urban, and the stream and river density.

**Table 1.** The top ranked first-order occupancy and detection models for the pine marten, the red squirrel and the grey squirrel. (For full model selection tables, see the electronic supplementary material, tables S1–S4.)

species	top occupancy model (*ψ*)	top detection model (*p*)
pine marten	*ψ*(broadleaf, conifer, built, year, latitude)	*p*(bait, occasion, previous, conifer, year)
red squirrel	*ψ*(built, conifer, year, latitude),	*p*(occasion, previous, broadleaf, people, year)
grey squirrel	*ψ*(broadleaf, conifer, built, year, latitude, longitude)	*p*(bait, occasion, previous, broadleaf, built, river, year)

The multi-species model allows formal investigation of how habitat mediates species interactions by specifying models for multiple pairwise interactions for each species pair simultaneously. Specifically, we were interested in examining how the probability of two species co-occurring at the same site was mediated by the two main habitat types for each of these forest dwelling species: broadleaf forest native woodland (BL) and non-native coniferous timber plantations (CP, [11]). For each species, we considered three possible second-order scenarios: (i) the *independence* hypothesis that the species occur independently of one another as a function of habitat covariates only; (ii) the *constant* hypothesis that species exhibit constant pairwise dependence that do not vary across space; and (iii) the *habitat* hypothesis that co-occurrence between the species varies as a function of habitat. We constructed a candidate model set with second-order models that represent the possible combinations of the three hypotheses for each species, resulting in a total of 27 models. Following the ‘natural parameter’ terminology of Rota *et al*. [21], parameters *f*_1_, *f*_2_ and *f*_3_ are the natural-scale first-order occupancy probabilities for pine marten, red squirrel and grey squirrel, and are described in the text above (see also table 1). The second-order models that describe how the interaction between species *i* and *j, f_i_*_,*j*_ depend on the hypothesis and are: *f_ij_* = 0, *f_ij_* = *β*_0_, and *f_ij_* = *β*_0_ + *β*_1_BL + *β*_2_CP for the *independence*, *constant* and *habitat* hypothesis, respectively. Here, *β*_0_ is the intercept and *β*_1_ and *β*_2_ are the estimated effects of broadleaf and coniferous covariates, respectively.

We fit the multi-species occupancy models in R v. 3.6 [28] using the package *unmarked* and the function *occuMulti()* for model fitting [29]. Given our multiple competing hypotheses, we used *AICcmodavg* for AIC-based model ranking [30], models with ΔAIC values < 5 when compared with the most parsimonious model are presented [26].

## 3. Results

Total effort for the three surveys over the 5-year period was 7286 sampling days (24 h periods) across 712 sites (2015 = 2631 at 332 sites; 2018 = 1845 at 173 sites; 2020 = 2881 at 207 sites). Over the course of 5 years, there were a total of 2452 independent detections of the three focal species, composed of 830 pine marten detections (2015, *n* = 214; 2018, *n* *=* 89, 2020 *n* = 527), 963 red squirrel detections (2015, *n* = 210; 2018, *n* = 263, 2020, *n* = 490) and 659 grey squirrel detections (2015, *n* = 332; 2018, *n* = 113; 2020, *n* = 214).

Using AIC to compare the multi-species models, we found clear evidence of interspecific dependence among all three species, and habitat mediation of coevolved but not novel predator–prey interactions (table 2). Specifically, the top model supported the hypotheses that the probability of coevolved pine marten and red squirrel co-occurrence depended on habitat, but that novel interactions between the native-invasive pairs (pine marten-grey squirrel and red squirrel-grey squirrel) were constant, i.e. were not mediated by habitat. All subsequent results are from the top model, apart from when explicitly stated otherwise. All values reported are mean estimates ± s.e.

**Table 2.** AIC model selection between the 27 *a priori* multi-species candidate models representing different hypotheses regarding the impacts of habitat on species interactions and their importance as drivers of occurrence and co-occurrence of the red squirrel, the pine marten and the grey squirrel. (Only models with ΔAIC values <5 are shown. *K*, number of parameters; AIC is the Akaike information criterion and *ω_i_* is the model weight. PM is pine marten, RS is red squirrel and GS is grey squirrel.)

model	*K*	−2 log likelihood	AIC	ΔAIC	*ω_i_*
*f*_PM−RS_(habitat), *f*_PM−GS_(constant), *f*_GS−RS_(constant)	41	−4474.39	9030.78	0	0.43
*f*_PM−RS_(habitat), *f*_PM−GS_(habitat), *f*_GS−RS_(constant)	43	−4472.98	9031.97	1.19	0.24
*f*_PM−RS_(constant), *f*_PM−GS_(constant), *f*_GS−RS_(constant)	39	−4477.82	9033.63	2.86	0.1
*f*_PM−RS_(habitat), *f*_PM−GS_(constant), *f*_GS−RS_(habitat)	43	−4473.98	9033.95	3.17	0.09
*f*_PM−RS_(constant), *f*_PM−GS_(habitat), *f*_GS−RS_(constant)	41	−4476.32	9034.64	3.87	0.06
*f*_PM−RS_(habitat), *f*_PM−GS_(habitat), *f*_GS−RS_(habitat)	45	−4472.78	9035.56	4.79	0.04

**Figure 1.**
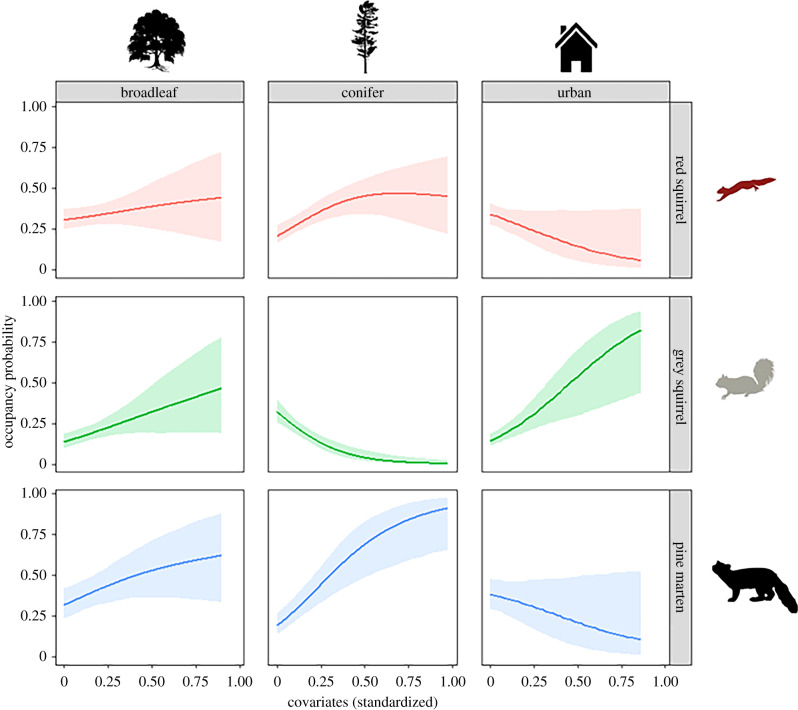
Marginal probability of occupancy for the red squirrel (red), grey squirrel (green), and pine marten (blue) as a function of proportion of broadleaf woodland, coniferous plantation and urban and suburban land use. (Online version in colour.

The probability of occupancy of both the pine marten and the red squirrel considerably increased across the 5-year period (electronic supplementary material, appendix S2, figure S2.1; pine marten from 0.27 ± 0.09 to 0.53 ± 0.11 and red squirrel from 0.27 ± 0.07 to 0.38 ± 0.05). The opposite was true for grey squirrels; their occupancy declined substantially from 0.23 ± 0.06 to 0.11 ± 0.04. Marginal probabilities of occupancy show that pine marten occurrence was positively associated with both broadleaf and mixed forests (*β* = 0.27 ± 0.15) and coniferous plantations (*β* = 0.96 ± 0.27), and negatively associated with urban and suburban areas (*β* = −0.30 ± 0.17; figure 1). Red squirrels showed a similar pattern: occupancy was positively associated with coniferous plantations (*β* = 0.50 ± 0.22) and negatively associated with urban and suburban areas (*β* = 0.32 ± 0.15; figure 1). Grey squirrel occurrence was positively related to urban and suburban areas (*β* = 0.44 ± 0.12) and broadleaf woodland (*β* = 0.43 ± 0.12), but negatively associated with conifer plantations (*β* = −0.56 ± 0.22; figure 1).

**Figure 2.**
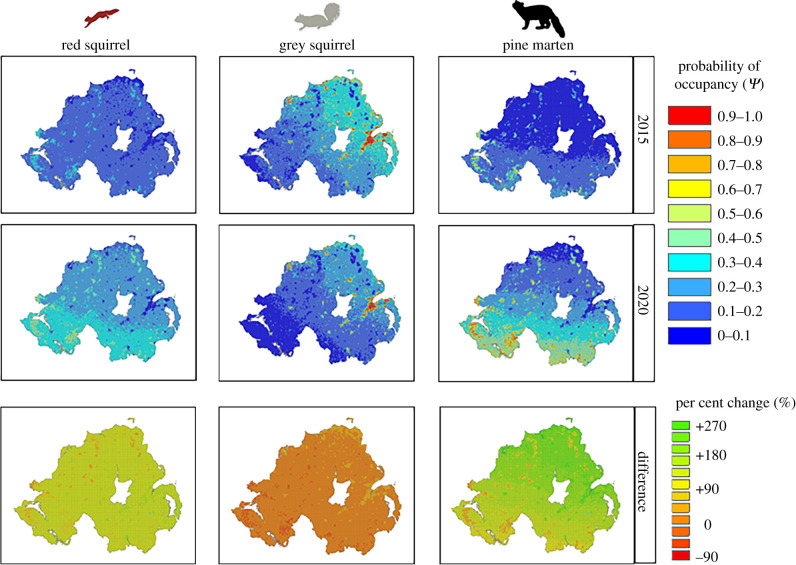
Predicted probability of occurrence across 14 401 km^2^ of Northern Ireland from 2015 to 2020 for the pine marten, grey squirrel, and red squirrel based on multi-species occupancy models applied to the entire 2015–2020 survey data (*n* = 706) predicting occupancy for each year of sampling or the specific land-cover covariates of each 1 km^2^ of the region and the per cent change in occupancy estimates from 2015 to 2020.

Spatially explicit predictions of occupancy over the 5 years show the rapid recovery of the pine marten, with the species now occurring throughout the region, although occupancy remains highest in the southwest and in forested areas (figure 2). Red squirrels have undergone a similar recovery in the same locations as the pine marten, with the mean occurrence increasing across the landscape but with highest probabilities of occupancy in the south and forested areas (figure 2). By contrast, grey squirrels have undergone declines and have gone from the most widespread of the three species to the most range restricted (figure 2).

**Figure 3.**
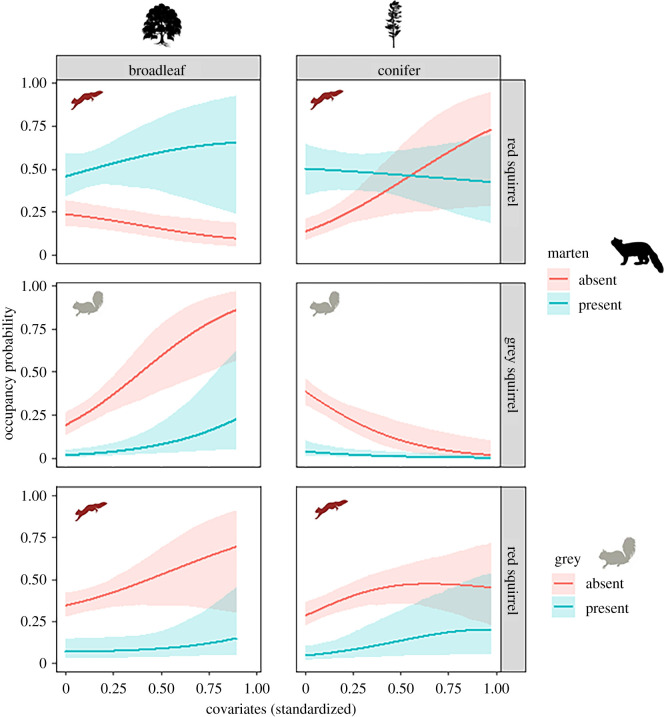
Occupancy probability of the red squirrel and the grey squirrel conditional on the presence (blue) and the absence (red) of their shared predator, the pine marten (top and centre panels) and the grey squirrel (bottom panels) in the two main habitat types available to the species; native broadleaf woodlands (left) and commercial conifer plantations (right). All variables not included in a panel are fixed at their observed means.

Credible intervals of the pairwise intercept parameters for species interactions (*β*_0_ from the second-order models) e.g. *f*_12_, *f*_13_ and *f*_23_ did not overlap 0, demonstrating statistical support for the integral role of the interspecific interactions driving the occurrence patterns of the three species across the landscape. Overall, pine marten and red squirrel were positively associated (*β*_0_ = 0.95 ± 0.33), there was a negative association between pine marten and grey squirrels (*β*_0_ = −2.24 ± 0.59), and likewise, grey squirrels and reds squirrels were negatively associated (*β*_0_ = −1.68 ± 0.47). The co-occurrence of the pine marten and the grey squirrel did not vary across habitats, with strong suppression of the grey squirrel by the native predator across the entire gradient of both forest habitat types (figure 3). This was also the case for the competitive interaction between the red squirrel and the grey squirrel, where the predicted occurrence of red squirrels remained close to zero in the presence of the invader, regardless of changing proportions of habitat composition (figure 3). In contrast with the naive pairs, the co-occurrence of the evolved predator–prey pairing of pine marten and red squirrel was mediated by habitat. While red squirrels were observed to be outcompeted and suppressed in broadleaf woodlands in the presence of grey squirrels, they reached high occupancy probabilities in broadleaf woodlands in the presence of their shared predator, the pine marten (figure 3; *β*_1_ = 0.16 ± 0.14). On the contrary however, this positive effect was reversed in conifer plantations, with pine marten presence having a negative effect on red squirrel occurrence as the proportion of commercial plantation increased (figure 3; *β*_2_ = −0.58 ± 0.29).

It is worth noting that the second highest ranked model, which had a weight of 0.24 (table 2), also included habitat effects for the pine marten and grey squirrel, but they were consistently negative across both habitats, with one of the effects overlapping zero and thus not significant (*β*_1_ = −58 ± 0.43, *β*_2_ = −0.49 ± 0.73). As such, the inferences drawn from the top model are qualitatively and quantitatively identical with the exception that slightly stronger suppression of grey squirrels is predicted in the second ranked model (see the electronic supplementary material, appendix S2, figures S2.2 and S2.3).

Finally, a learned response explained the most variation in detection probability of all three species with detection probability increasing after an initial detection (pine marten *β* = 0.73 ± 0.08, red squirrel *β* = 0.74 ± 0.07 and grey squirrel *β* = 0.46 ± 0.01). The detection probability of pine martens and grey squirrels also varied as a function of the bait used at the feeders (pine marten *β* = 0.41 ± 0.06, grey squirrel *β* = 0.21 ± 0.07; detection of both decreased in the absence of peanuts); however, this was not observed in red squirrels (electronic supplementary material, appendix S2, figure S2.3). The detection probability of the three species also varied as a function of a small number of environmental (broadleaf, conifer, river) and human disturbance (people per km^2^ and urban/suburban) covariates but only to a small degree relative to the effect of a learnt response and bait (electronic supplementary material, appendix S1 and table S1).

## 4. Discussion

We provide empirical evidence that habitat modifies the direction and strength of coevolved predator–prey interactions, but not interactions between evolutionarily naive species pairs. Overall, the occurrence of the native red squirrel was higher in the presence of the native pine marten, an effect that increased in native broadleaf woodlands but was reversed in non-native commercial conifer plantations. In fact, in these simplified conifer landscapes, the presence of the pine marten reduced the likelihood of red squirrel occurrence. By contrast, neither the direction nor strength of interactions between the novel pairings was influenced by habitat. First, pine martens suppressed grey squirrels regardless of habitat, directly supporting the hypothesis that the restoration and recovery of native predator populations can provide highly valuable biological control of established invasive species, even in highly human-modified landscapes. Second, grey squirrels suppressed red squirrels regardless of habitat, directly supporting the disease-mediated competition hypothesis. Combined, these results demonstrate that while habitat modification has the potential to disrupt established predator–prey interactions between coevolved species, these negative effects are far outweighed by the benefits of competitive release where a dominant invasive competitor is controlled by the recovering predator.

While habitat complexity has been shown to reduce attack rates and foraging efficiencies of predators [31,32], we demonstrate clear differences in the effects of habitat on predator–prey interactions between native and invasive species. Habitat-specific differences in the interactions between a shared native predator and native and invasive prey could stem from the degree of naivety to the predation threat [19], whereby coevolved prey have developed appropriate and effective anti-predator behaviours, which remain absent in the invasive species. This disparity in anti-predator behaviours may result in the native prey only being targeted in simplified habitats where alternate prey is limited. Whereas, the naive invasive analogue, being more susceptible to predation, remains a highly profitable prey item regardless of local habitat complexity. The native prey is, however, suppressed in the presence of the native predator in the habitat where the invasive competitor does not occur, suggesting an alternate mechanism. In the absence of pine martens, red squirrels are thought to be able to persist in conifer plantations owing to a competitive advantage over grey squirrels [20]. Thus, in conifer plantations, the red squirrel does not benefit from competitive release from grey squirrels following pine marten recovery but is subject to predation by the shared predator [33]. How habitat mediates the impacts of a recovering predator on a native prey population appears to be underpinned by additional indirect interactions from an invasive competitor.

These results are of global significance when considering the benefits of predator recovery [10,34], and mounting calls for reintroductions of carnivores to their previous ranges to restore ecosystem function [35,36]. We highlight the need to further understand how human-modified landscapes may affect interactions between recovering carnivore populations and native species in the absence of invasive species, to better predict the impacts of such recoveries. Our research demonstrates that the indirect benefit of controlling an invasive competitor is far stronger than the negative effect of direct predation. The occurrence of a native prey species increases on average across the landscape following the return of one of its key predators [33,37,38]. Thus, native predator recovery benefits native prey populations when it results in a release from competition with invasive counterparts. This process shares the same mechanistic underpinning and consequences recently observed in the recovery of native northern spotted owls (*Strix occidentalis caurina)* following large-scale human control efforts of the invasive barred owl (*Strix varia*, [39]).

Pine marten occupancy more than doubled over a 5 year period from 2015 to 2020, while red squirrel occurrence increased by approximately one-third in the same period. Moreover, the recoveries of both species were also geographically coupled (figure 2). In contrast with the native species, the occupancy of the invasive grey squirrel more than halved from 2015 to 2020 and declined in the same areas pine martens recovered. Our results far exceed predictions from a single survey [11]. This has critical implications for the management of invasive species and the monitoring of recovering predator populations in the future. Repeated surveys through time are necessary to ensure that predictions are robust, and not an ephemeral by-product resulting from the temporality of sampling.

While our approach is not strictly experimental, we couple probabilistic methods that explicitly account for imperfect detection with a large and representative sample, thus meeting the statistical rigour required to inform policy on wildlife conservation and management. Our multi-species approach provides key insights into factors driving the occurrence and interactions of a complex and conservationally important interaction network which were not otherwise evident. For example, previous research has suggested that interactions between red and grey squirrels depend on habitat alone, with predictions ranging from complete extirpation of the native inferior competitor in native broadleaf woodlands to the persistence of the inferior native competitor in large commercial conifer plantations [11,20]. This has led to recommendations that national conservation strategies for red squirrels should focus on the planting of commercial conifer plantations as opposed to native broadleaf forests, where grey squirrels have a competitive advantage [11,20]. Our results suggest that such management strategies could undermine ongoing red squirrel recovery efforts, with consequences likely antithetical to their intention. When accounting for additional actors, and the mediating role of habitat, we observed a reduction in the occurrence of red squirrels in large structurally simple conifer plantations where pine martens were present. Such commercial plantations constitute the majority of Ireland and Scotland's forest cover, where they continue to be planted under the guise of saving the red squirrel [20]. Our results suggest that landscape management strategies for red squirrel conservation would be best focused on planting native broadleaf woodlands alongside continued pine marten restoration efforts.

Here, we show that in the presence of invasive species, human modification of habitats does not alter the beneficial impacts of native predator recovery on native prey species through competitive release. However, in the absence of invasive competitors, habitat composition has the potential to benefit, or alternatively, to have deleterious impacts on native prey populations following predator recovery. We highlight the necessity of including interspecific interactions in models predicting the occurrence of species for management plans and conservation strategies. Conservation strategies that fail to consider the interactions between environmental conditions and interspecific interactions are probably subject to biases that may, in turn, lead to misguided, and potentially disastrous wildlife management strategies. We conclude that while predator restoration is a vital conservation strategy in the face of increasing invasions and declining global diversity, it should be in conjunction with efforts to restore and maintain a range of natural, structurally complex habitats.

## Data accessibility

All data (detection-non detection data for each species from the three surveys and associated site level covariate data) are available from the Dryad Digital Repository: https://doi.org/10.5061/dryad.r4xgxd2dv [40].
